# Paradise Lost *Eden with Leish and Fer-de-Lance*

**DOI:** 10.4269/ajtmh.17-0493

**Published:** 2017-08-02

**Authors:** Claire Panosian Dunavan

**Affiliations:** University of California Los Angeles, California

In June 2015, Douglas Preston—amateur archaeologist, National Geographic journalist, and author of best-selling thrillers—entered an infusion suite at the National Institutes of Health sporting an “oozing crater, fiery red and disgusting to look at” on his left upper arm. Ensconced in the room's weirdly large chairs, the 61-year-old breathed deep, ready to receive his initial hit of amphotericin B. “It was a total anticlimax,” Preston later reported in “The Lost City of the Monkey God,” a fast-paced, often harrowing saga of a recent expedition to Honduras. Published this year, the book describes adventurers, scientists, photographers, soldiers, and ex-commandos who found tantalizing traces of a lost civilization in a jungle called La Mosquitia. Roughly two thirds of the entourage, more than thirty people, also contracted leishmaniasis.

In January 2017, I learned of “The Lost City of the Monkey God” through an e-mail. Later that morning, could I travel to a nearby studio to provide expert perspective on leishmaniasis? a TV producer gamely asked. Well, no, I replied. But by now I was sufficiently intrigued to request the book from my library. Once I read the chapter in which Doctors Ted Nash and Elise O’Connell started to treat Preston’s *Leishmania brasiliensis* infection, and the author also met NIH researcher David Sacks and inspected colonies of sandflies and leish-infected mice, I privately thanked “Inside Edition” for leading me to a perfect candidate for The Tropical Bookshelf.

“Danger porn” is a term some have applied to Preston’s spellbinder, which certainly offers its share of edge-of-the-seat scares. But before considering Honduras’s bounty of vipers, jaguars, and quicksand, would you like to learn more about Theodore Morde, a Massachusetts-born journalist-cum-deceiver who first covered the Spanish Civil War, then traveled to Mosquitia to slake his lust for gold at the same time cabling fake accounts of a legendary *Ciudad Blanca* (White City)? If your answer is yes, Preston delivers. Then there’s 19^th^ century banana baron Samuel Zemurray, AKA “Sam the Banana Man,” a one-time pushcart seller who eventually bilked both J. P. Morgan and the U.S. government. Long before the country became famous for narco-trafficking, Preston coolly reminds us, “the fruit companies left a dark colonist legacy that has hung like a miasma over Honduras ever since.”

Back to the 21^st^ century. If the dense Mosquitia rainforest is Eden in ruins, there must be a snake, right? Oh boy, is there a snake. A fer-de-lance appears the same night Preston and company set up camp. Preston is the first to spy the savage viper, its eyes fixed, its tongue flickering. One-time jungle commando Andrew Wood then brandishes a machete and lops off the branch of a tree, swiftly forming a long, forked pole.

*“Keeping the neck pinned with the stick and his left hand,” *Preston continues,* “Woody crouched and seized it behind the head with his right hand. The snake’s body, thick as his arm, slammed against his legs, its dazzling snow-white mouth gaping wide, unsheathing inch-and-a-quarter-long fangs that pumped out streams of pale yellow liquid. As its head lashed back and forth, straining to sink its fangs into Woody’s fist, it expelled poison all over the back of his hand, causing his skin to bubble.”*

Following its decapitation, the snake’s head and neck continue to wriggle in one direction, while its muscular body crawls off in another. Egad.

Although adventure is the signature hook of “The Lost City,” a near-lyrical passage marks Preston’s first sighting of a twenty-square-mile, unexplored tract called “Target One.” The aging Cessna from which the author stares is equipped with LIDAR (the acronym stands for Light Detection and Ranging, expensive laser-based technology first used to map the surface of the moon) but hardly inspires confidence. Nonetheless, Preston desperately wants on. For ASTMH members who have braved similarly rustic aircraft in distant realms, the next excerpts may bring back memories:

“As I crawled on board, my dismay deepened. The interior of the Cessna, once a rich velvetized fabric in burgundy, was now worn, greasy, and faded: much of the inside appeared to be held together with duct tape. It smelled of Eau de Old Car. Parts of the plane had been sealed with acrylic caulk, now peeling out in strings. As I tried to maneuver around the giant lidar box into the microspace provided, I bumped my elbow into a panel which fell off.”

“I jammed myself behind the lidar box: no seat, my knees in my mouth. Juan Carlos was right in front of me. He was concerned about how I would fare; I sense he was worried I might get airsick and vomit down the back of his neck… The A/C on the plane was broken, he said; we would be sealed up in a metal tube, flying in full sun. The plane had no bathroom. If you had to go, you went in your pants. I tried to assure him I would be an exemplary passenger.”

“Through it all, I peered out the window, transfixed. I can scarcely find the word to describe the opulence of the rainforest that unrolled below us. The tree crowns were packed together like puffballs, displaying every possible hue, tint and shade of green. Chartreuse, emerald, lime, aquamarine, teal, bottle, glaucous, asparagus, olive, celadon, jade, malachite—mere words are inadequate to express the chromatic infinities.”

Later we come to the book’s unexpected switchback: the point at which the adventurers have returned home and some of their lingering bug bites begin to morph into typical nodules, sores, and crusts of cutaneous leishmaniasis, a neglected disease which, every year, still afflicts one to two million people worldwide. Growing increasingly apprehensive about the possible metastatic spread of certain New World species, Preston first reaches out to Ravi Durvasala (a specialist in Old World cutaneous leishmaniasis) in Albuquerque, New Mexico, then to Tom Nutman and Ted Nash at the NIH’s Laboratory for Parasitic Diseases. By then, Nash had already biopsied, cultured, and sequenced the *L. brasiliensis* strain of a fellow Mosquitia traveler. Nash would eventually see five or six of the venturesome crew, he later told me, wryly adding: “I enjoyed taking care of these guys—they were great patients, kind of like Peace Corps volunteers. Of course it’s always tricky treating multiple people who are all yapping about each other at the same time they’re incredibly bonded.”

Derring-do, drop-dead primeval beauty, and “white leprosy” notwithstanding, I found Preston’s closing chapters the most powerful. What actually happened to the inhabitants of Mosquitia? Today, while archaeologists continue to interrogate clues buried in forest, thicket, and vine, we can only guess the cause of their tragic demise. Most likely, Preston surmises, a newly-introduced virus took them out, as it did so many other indigenous Americans of that era. To evoke this cataclysm, he quotes from a manuscript found in 1844 in a remote Guatemalan convent. Written in the Mayan dialect of Cakchiquel, its original narrator was just a teen-aged boy when his world forever changed.

It happened during the twenty-fifth year [1520] the plague began, oh my sons! First they became ill of a cough, they suffered from nosebleeds and illness of the bladder. It was truly terrible, the number of dead there were in that period. The prince Vakaki Ahmak died then. Little by little heavy shadows and black night enveloped our fathers and grandfathers and us also, oh my sons!... Great was the stench of the dead. After our father and grandfathers succumbed, half of the people fled to the fields. The dogs and the vultures devoured the bodies. The mortality was terrible… So it was that we became orphans, oh my sons! So we became when we were young. All of us were thus. We were born to die!

Across the centuries, humanity keens. Snakes and sandflies are perilous, but what can be worse than an unseen, mass assassin?

Preston also shares a second contemporaneous account, originally written in Yucatec Mayan, which is at once touching, poetic, and far more explicit in linking European conquest with Native death and destruction.

“There was then no sickness; they had no aching bones; they had then no high fever; they then had no smallpox; no stomach pains; no consumption… At that time people stood erect. But then the foreigners arrived and everything fell apart. They brought fear, and they came to wither the flowers.”

## INTERVIEW WITH DOUGLAS PRESTON

### Let’s start with your life as an explorer and a writer. For example, what drives your passion for adventure as well as your prolific output of fiction and non-fiction?

Basically, it boils down to this. I’m not really interested in normal people doing ordinary things. I’ve always been attracted to exploring how human beings react when they are placed in extraordinary or dangerous or life-threatening circumstances. That’s what my nonfiction is about and it’s also what my fiction is about. In nonfiction, I deal with exploration. In fiction, I write the thriller.

### Looking back, did you become personally obsessed with finding the Lost City of the Monkey God, or were you merely dogged in your pursuit of a great story? Also, what are some of the most haunting memories or epiphanies stemming from your 2015 trip to Honduras?

Well, it’s a good question. You know, I think what started off as a dogged pursuit of a story became an obsession—or maybe a quasi-obsession. Because the more I looked at the story and the more I researched it, the more I realized that there was a lot of solid evidence suggesting truth behind this legend. In fact, almost all of the archaeological sites that have been found in Central America have been found by archaeologists who were led by indigenous people or by stories or legends about a city somewhere in the jungle. In the end, what we discovered was not just a lost city but a lost civilization that had once been great but was almost unknown because it was totally overshadowed by the Maya.

The one moment that has really stayed with me is when we went in on the ground in 2015. On the second day, we discovered an immense cache of objects left at the base of a pyramid. And the first thing I saw was a jaguar head thrusting out of the ground. It was a major moment because, up to that point, it had all been theoretical. I mean, you see giant mounds and all kinds of evidence of occupation. But suddenly I felt like I was face to face with these long-lost people. The [jaguar] image had such power, such confidence, such vividness and artistic sophistication. That really brought home that this was a civilization of great interest and complexity that had somehow grown up in a very hostile jungle.

### Please reflect on the quote which describes Native American life pre- and post- European contact and ends with the sentence: “They brought fear, and they came to take away the flowers.”

For 15,000 or 20,000 years, ever since the first humans crossed the Bering land bridge and populated the Americas, it was a biological time bomb waiting to happen. These two populations had very different disease trajectories. When they finally met–and it was inevitable that it would happen at some point–there was going to be this absolute catastrophe. There was no preventing it. I mean, even if the Europeans or Spanish hadn’t [arrived]–well, the Africans or Asians would have. The same result would have occurred, which was an absolutely horrific series of pandemics that probably wiped out 90% of the population of the New World. What I tried in my book, as hard as I could, was to really imagine what it would have been like to experience a pandemic where 90% of the people die.

I’ve always been interested in the Black Death and how it affected Europe and may have even triggered the Renaissance. I’m sure you know all the theories. But the Black Death at its worst caused 30% mortality except for a few pockets with 60% mortality. The 90% mortality isn’t just quantitatively more, it’s a qualitative jump because it destroys entire civilizations, languages, cultures, life cycles. It takes away everything you have.

### What can you tell us about your own fears before entering La Mosquitia. At the beginning of your book, you describe a daunting orientation cataloging all kinds of dangers.

To be honest, I tend to be an optimist and to dismiss these sorts of things. I have spent most of my life backpacking in wilderness areas and climbing mountains and surviving in adverse conditions. So I thought to myself: well, this catalog of horrors is meant for people who have never been in a wilderness before. I’m going to be fine. Then when I got there and saw that it was not an exaggeration—that it was an extremely challenging and dangerous area—I thought, well, look, you know what? This is a place where human beings have not been in probably 500 years and we are not welcome here.

It’s good for humans to be utterly humbled by nature every once in a while. That’s exactly what happened to us in this environment.

### Would you do it again, knowing what you now know? And would you go back?

Even if I knew I was going to get leish, I would still do it again. And I would also go back if they found something new. Here’s a bit of information that has not been published. Conservation International is down there and have found a species of bat that only lives in deep caves. One thing we know about this culture is that they bury their dead in caves. If these caves are a necropolis, there would be grave goods and all kinds of fascinating stuff that would open the door on this culture in a way that has never happened before.

### I’m sure lots of people also ask you about your close call with the fer-de-lance.

You know, I’ve had many encounters with poisonous snakes in my life. With rattlesnakes, I’ve been struck on my boot; then they bounced off, thank God. Anyway, I’m acutely aware of snakes and I actually like them. And a lot of snakes, really, are terrified—they just want to get away, even if they hit you and bite you. Fer-de-lance are different; they can strike and will often hit again. When I saw that fer-de-lance coiled in striking position, tracking me—I walked by it twice–I thought: wow, they’re very aggressive. Really, really bad. When I saw that snake, I was sobered.

The British SAS guy who killed it, at the end when he’s washing the venom off his hands, said: “Nothing like that to concentrate the mind, is there?” If it hadn’t been such a scary moment, it would have been funny.

### What was scarier, the vipers or the possible face-eating complication of mucocutaneous leishmaniasis?

I’ll be honest with you: the vipers were a lot scarier.

Addendum: According to current estimates, snakebites kill more than 100,000 people per year. In an effort to reduce that number, WHO recently added venomous snake bites to its list of neglected tropical diseases.

### Finally, what impressed you most about your journey to diagnosis and treatment culminating in your care at NIH?

I just want to say how impressed I was with my experience at the NIH and its staff, doctors, and researchers, in particular Ted Nash, Elise O’Connell, and David Sacks. At NIH, our taxpayer dollars are being incredibly well spent for the benefit of the American people and the world. It’s a perfect example of government doing something so much better than private industry. Because government doesn’t have the financial and profit requirement that industry has… it can fund all kinds of far-reaching and important basic research that private industry can’t.

